# Effect of Diabetes Mellitus on Survival in Patients with Pancreatic Cancer: A Systematic Review and Meta-analysis

**DOI:** 10.1038/srep17102

**Published:** 2015-11-24

**Authors:** Yixiang Mao, Min Tao, Xiaoyan Jia, Hong Xu, Kai Chen, Hongwei Tang, Donghui Li

**Affiliations:** 1 Department of Oncology, The First Affiliated Hospital of Soochow University, Suzhou, China; 2 Department of Gastrointestinal Medical Oncology, The University of Texas MD Anderson Cancer Center, Houston, Texas, USA; 3 Department of Medical Oncology, Fudan University Shanghai Cancer Center, Shanghai, China; 4 Jiangsu Institute of Clinical Immunology, Suzhou, China

## Abstract

Concurrent diabetes has been linked with an increased risk of death in many cancers, but findings in pancreatic cancer have been inconsistent. We performed a systematic review and meta-analysis to assess the effect of diabetes on survival in patients with pancreatic cancer. Of 4, 463 original articles, 41 were included in the review; 29 studies with 33 risk estimates were included in the meta-analysis. In the overall comparison of patients with pancreatic cancer and diabetes with their nondiabetic counterparts, the former had significantly higher all-cause mortality (pooled HR: 1.13; 95% CI: 1.04–1.22). Subgroup analyses showed that diabetes was associated with poor survival in patients with resectable disease (HR: 1.37; 95% CI: 1.15–1.63) but not in those with unresectable disease (HR: 1.07; 95% CI: 0.89–1.29). The HR (95% CI) was 1.52 (1.20–1.93) for patients with new-onset diabetes (≤2 years of diabetes duration) and 1.22 (0.83–1.80) for those with longstanding diabetes (>2 years). Diabetes was associated with higher mortality overall in patients with pancreatic cancer. The effect of diabetes on overall survival was associated with the stages of tumor and the duration of diabetes.

Diabetes mellitus (DM), or impaired glucose tolerance, is concurrently present in 50–80% of patients with pancreatic cancer (PC), one of the most rapidly fatal malignancies. DM is a known risk factor for PC^1^,^2^,^3^; furthermore, new-onset DM could be an early sign of PC^4^, resulting from insulin resistance induced by a paraneoplastic syndrome^5^ or pancreatic β-cell dysfunction^6^.

Increasing evidence suggests that patients with colorectal, breast, liver, endometrial, and gastric cancers and leukemia^7^,^8^ who also have DM are at increased risk of cancer recurrence, cancer-related death, and death from any cause. However, whether and how the concurrent DM may affect clinical outcome in PC has not been determined, and available information on this topic is limited and inconsistent. Some studies found that DM did not have a significant effect on overall survival (OS) duration^9^,^10^,^11^,^12^,^13^,^14^,^15^,^16^,^17^,^18^,^19^,^20^,^21^,^22^,^23^, whereas others found that DM was associated with significantly reduced survival duration^24^,^25^,^26^,^27^,^28^,^29^,^30^,^31^,^32^,^33^,^34^. These inconsistent findings may be partially explained by small sample sizes and/or by not adjusting for body mass index (BMI), disease stage, and other possible confounders^35^. Understanding the prognostic relevance of DM in PC may lead to better clinical management of this devastating disease. We therefore conducted a systematic review and meta-analysis to illustrate the association between preexisting DM and mortality in patients with PC.

## Methods

### Data sources and searches

We searched PubMed, Embase, and Web of Science (TS) databases from their inception to September 2, 2014, for articles evaluating the association between DM and outcome in PC, including survival, stage at diagnosis, and treatment choice. Our overall search strategy included terms for diabetes (e.g., “diabetes,” “glucose intolerance,” and “hyperglycemia”), pancreatic cancer (e.g., “cancer,” “carcinoma,” “adenocarcinoma,” “pancreatic,” and “pancreas”), and prognosis (e.g., “prognosis,” “survival,” and “mortality”). We also searched the references of included articles. No language or publication type restrictions were imposed (Supplementary Table 1).

### Study selection

**Figure 1:**
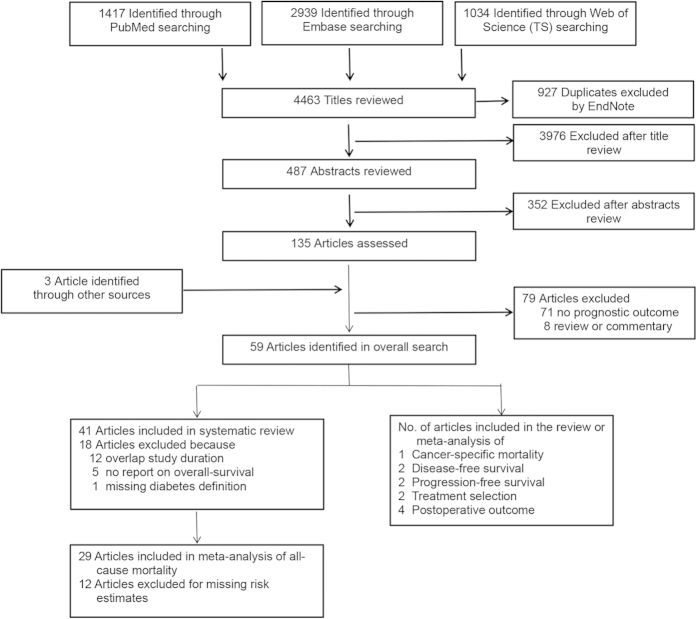
Flowchart of study selection.

Our overall search targeted articles describing studies that met the following three criteria: 1) evaluated any prognostic outcome by DM or glycemic status; 2) evaluated a PC patient population; and 3) contained original data analysis. We included studies evaluating type 1 and/or type 2 DM. To avoid overlapping patient populations, we compared data on recruitment years, data source, and geographic location. Publications with duplicate datasets were triaged by keeping the most recent one, the one with the larger study population, or the one with multivariate-adjusted estimates. Articles that met the above three criteria and reported all-cause mortality or OS were included in our systematic review (Fig. 1). To be included in our meta-analysis, articles had to report a risk estimate (e.g., hazard ratio [HR]) relating preexisting DM to subsequent death by using survival analysis regression models, with an estimate of precision, such as standard error (SE) or 95% confidence interval (CI). Articles with missing risk estimates were also included in the meta-analysis if the risk estimates were generated by author contact. Of the 14 authors contacted, 7 responded, 6 provided additional information. We also conducted a systematic review or meta-analyses on long-term, cancer-specific mortality, disease-free survival (DFS), progression-free survival (PFS), postoperative death rate, and choice of cancer treatment in studies with the relevant information including studies that were excluded from the general review and meta-analysis because of overlapping study durations^36^,^37^,^38^.

### Data extraction and quality assessment

Each article was abstracted by one author and reviewed by the second author for accuracy. Any disagreement was resolved by consensus. If several estimates were reported in the same article, we chose the most fully adjusted estimate (i.e., multivariate regression was selected over univariate regression, which was selected over unadjusted Kaplan-Meier analysis). If an article reported multiple estimates by subgroup only, these estimates were entered separately into our relevant meta-analysis dataset.

We also extracted information on key indicators of study quality with use of Meta-analysis of Observational Studies in Epidemiology (MOOSE) standards^39^ for reporting of meta-analyses of observational studies. From each study, we chose the risk estimates that represented the greatest degree of control for potential confounders. Quality was assessed by using elements of the Strengthening the Reporting of Observational Studies in Epidemiology (STROBE) statement^40^.

### Data analyses

The results of the systematic review were summarized qualitatively. The null hypothesis of “no additional mortality risk in cancer patients with preexisting DM” was tested with use of a nonparametric sign test.

For the meta-analysis, *P* values quoted at less than the specified threshold were assumed to be at the threshold, resulting in a conservative estimate of the significance level. *I*^2^ and Cochran *Q* estimates were performed in a heterogeneity assessment^41^. A *I*^2^ value of >50% or a *P* value of less than 0.1 represented significant heterogeneity. A DerSimonian-Laird random-effects model (D + L) was used to calculate the pooled HR. Otherwise, an inverse variance fixed-effects model (I-V) was selected. The meta-analysis was performed with use of Stata version 12.0 software (Stata Corp, College Station, TX).

To assess the impact of study quality, we conducted sensitivity analyses. We considered studies to be of higher quality and calculated separate pooled HRs if they were population-based (n = 4) or were adapted from full articles (n = 28), with estimates adjusted for confounders (n = 23) and DM evaluated as the primary exposure variable (n = 10). Publication bias was evaluated by using Begg’s funnel plot. We performed the Duval and Tweedie nonparametric trim and fill procedure^42^ to further assess potential effects of publication bias. This method considers the possibility of hypothetical missing studies, imputes their HRs, and recalculates a pooled estimate. For all tests, a *P* value (two-sided) of less than 0.05 was considered statistically significant.

## Results

Of the 4, 463 titles identified, 487 abstracts and 135 resulting full articles were reviewed to determine their eligibility (Fig. 1). Three additional articles were identified by searching references^9^. Of these 138 articles, 59 addressed the effect of DM on PC outcome. Eighteen of the 59 articles were excluded from the review for overlapping study duration, lack of definition for DM, or lack of focus on OS. As a result, 41 articles were included in the systematic review of the association of preexisting DM with long-term, all-cause mortality. Twenty-nine of these 41 articles with 33 risk estimates were included in the meta-analysis (Fig. 1)^9^,^10^,^11^,^12^,^13^,^14^,^15^,^16^,^18^,^20^,^21^,^22^,^23^,^24^,^25^,^26^,^27^,^28^,^29^,^30^,^31^,^43^,^44^,^45^,^46^,^47^,^48^,^49^,^50^. Descriptive data for studies included are listed in Table 1 and Supplementary Table 2.

### Description of studies

Forty-one studies had been conducted in the United States (n = 17), Europe (n = 13), Asia (n = 10), and Canada (n = 1). Sample sizes ranged from 21 to 22, 439 with a median of 367. Across the 41 studies that reported the number of participants with DM, the overall prevalence of DM was 35.7% (range, 9.7%–54.9%). Across the 34 studies that reported participant sex, 64.8% of the study population was male. Reporting of age and follow-up time varied widely across studies.

Survival analyses reported various outcomes, including cumulative one-year mortality rates, OS, DFS, and PFS. The studies used a variety of analytic techniques including 9 studies using Kaplan-Meier survival analysis only, 32 using Cox proportional hazards regression analysis (only 29 had exact HRs and 95% CIs), which were included in the following meta-analysis (Supplementary Table 3). The time origin for survival analysis was generally the time of cancer diagnosis, except in the case of treatment or surgical cohorts, for which the time of origin was the beginning of treatment or the date of tumor resection. Most studies were clinic-based design and 4 studies were population-based cohorts (Supplementary Table 3).

### Systematic review of evidence

The best evidence from each study is summarized here. Of 9 studies using Kaplan-Meier survival curves with the log-rank tests, DM was associated with decreased survival in 3 studies^33^,^34^,^51^ and 4 studies^19^,^52^,^53^,^54^ with and without statistical significance, respectively. DM was associated with nonsignificantly increased survival in 2 studies^55^,^56^.

Seven studies provided 9 crude HRs of death for preexisting DM including 3 nonsignificantly decreased risk^9^,^45^ and 6 null effect^13^,^15^,^18^,^44^,^57^. Of the 25 studies with multivariate HRs of death for DM, 12 reported significantly increased risk^24^,^25^,^26^,^27^,^28^,^29^,^30^,^31^,^32^,^47^,^48^,^49^, 2 reported nonsignificantly increased risk^11^,^16^, 2 had significantly decreased risk^46^,^50^, and 9 had null effect^10^,^12^,^14^,^20^,^21^,^22^,^43^,^54^,^58^.

Overall, DM was associated with increased risk of death in 25 estimates, decreased risk of death in 4 studies, and null effect in 16 estimates. The nonparametric sign test rejected the null hypothesis of equal mortality in patients with and without preexisting DM (*P* < 0.001).

### Meta-analysis on DM and all-cause mortality

**Figure 2:**
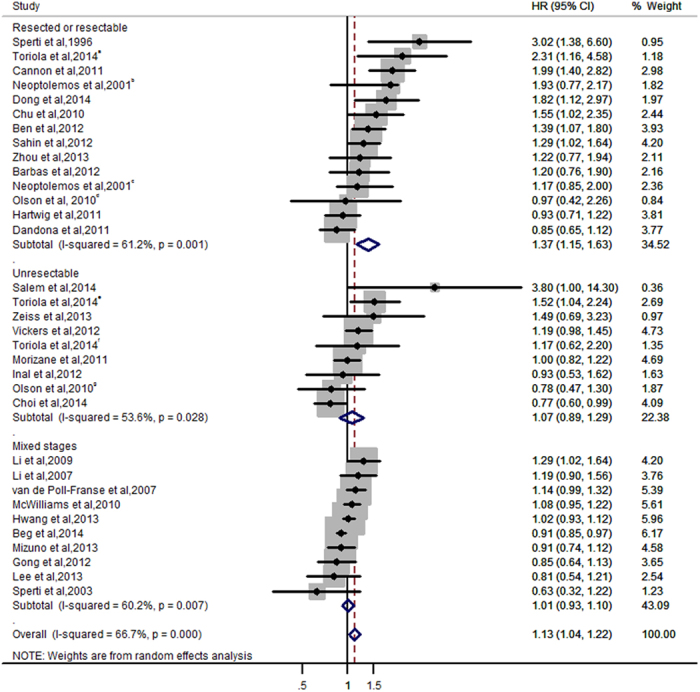
Meta-analysis and pooled hazard ratio of long-term, all-cause mortality in 29 studies comparing PC patients with and without preexisting DM. The 29 studies provided 33 estimates. Weights are from random-effects analysis. Data markers are proportional to study sample sizes. CI indicates confidence interval. Squares indicate relative risk in each study. The square size is proportional to the weight of the corresponding study in the meta-analysis; the length of the horizontal lines represents the 95% CI. The unshaded diamond indicates the pooled relative risk and 95% CI.

The 29 studies in the meta-analysis reported both risk (HR) and precision (95% CI). The descriptive data, adjustment or restriction variables, and major findings from each study are described in Table 1. The results of the meta-analysis are shown in Fig. 2. Preexisting DM was associated with a 13% increased risk of death from all causes in PC patients (HR, 1.13; 95% CI, 1.04–1.22). The pooled HR (95% CI) was 1.37 (1.15–1.63) from 13 studies conducted in patients with resectable disease, 1.07 (0.89–1.29) from 8 studies in unresectable disease, and 1.01 (0.93–1.10) from 10 studies in those with all-stage diseases. The Begg’s test and Duval and Tweedie trim and fill procedure showed no significant risk of publication bias (Begg’s test *P* = 0.14; Duvall and Tweedie adjusted HR: 1.10; 95% CI: 1.01–1.20; number of imputed studies = 3) (Supplementary Fig. 1).

### Sensitivity analyses

Considering the large variations in the covariates included in each study (Table 1), we conducted a sensitivity analysis to confirm robustness (Table 2). Risk estimates from higher-quality studies were similar to the overall estimate. The pooled risk estimate (HR [95% CI]) was 1.21 (1.06–1.39) for studies that took DM as the primary exposure variable. Studies with any adjustments had a pooled HR (95% CI) of 1.13 (1.03–1.24). More specifically, the above pooled HRs (95% CIs) after adjusting for age, BMI, and disease stage were 1.08 (0.98–1.20), 1.17 (0.98–1.39), and 1.09 (0.95–1.26), respectively. The risk estimates did not vary by publication types. The HR (95% CI) was 1.12 (1.03–1.21) for full articles.

Analysis of influence revealed that the risk of all-cause mortality among patients with PC and DM remained significant with the omission of each study in turn. Omission of the study by Cannon *et al.*^28^ resulted in the lowest pooled estimate (HR: 1.10; 95% CI: 1.02–1.19); omission of the study by Choi *et al.*^46^ resulted in the highest pooled estimate (HR: 1.14; 95% CI: 1.05–1.24).

### DM and cancer-specific mortality

One study^36^ provided adjusted HRs of cancer-specific death in patients who had undergone resection. It showed that patients with DM had a significantly higher risk of cancer-specific mortality compared with their non-DM counterparts (HR: 1.37; 95% CI: 1.00–1.89).

### Duration of DM and all-cause mortality

Six studies^20^,^27^,^30^,^33^,^37^,^52^ evaluated the association between duration of DM and OS of PC patients. In most studies, two years of diabetes duration was used as the cutoff for defining new-onset and longstanding DM. One study^33^ demonstrated that both new-onset and longstanding DM were associated with shorter survival by log-rank test. Three studies^20^,^37^,^52^ did not find a correlation between new-onset DM and OS in PC patients. Two studies^27^,^30^ indicated that OS duration in PC patients with new-onset DM, but not that in patients with longstanding DM, was significantly shorter than was OS in patients without DM. A meta-analysis of the two studies^27^,^30^ with risk estimates revealed new-onset DM as a significant prognostic factor (HR: 1.52; 95% CI: 1.20–1.93). We did not detect any significant heterogeneity (I^2^ = 0%, Q = 0.48; *P* = 0.49). However, the same prognostic value was not found in longstanding DM (HR: 1.22; 95% CI: 0.83–1.80) (Supplementary Fig. 2).

### DM and DFS, PFS

Two studies^28^,^38^ showed that having DM before undergoing tumor resection was independently associated with poor DFS (pooled HR: 1.54; 95% CI: 1.28–1.85) as well as poor OS after adjusting confounders. No significant heterogeneity was detected in the meta-analysis (I^2^ = 14.3%, Q = 1.17; *P* = 0.28) (Supplementary Fig. 3). Moreover, two studies^17^,^18^ found that in patients with advanced PC who were receiving systemic chemotherapy, PFS did not differ between those with and without DM.

### DM and treatment selection, postoperative mortality

No significant difference was found in the percentage of surgery between patients with and without DM, although DM patients were more likely recommended for resection^27^,^34^. Patients with DM had a higher likelihood of developing fistulas and acute kidney injury than did those without DM, but overall complication and severity did not differ between them^59^. The relation between DM and a higher postoperative mortality was not conclusive^59^,^60^,^61^,^62^.

## Discussion

Our study demonstrated that preexisting DM in PC patients, compared with their non-DM counterparts, was associated with increased risk of all-cause mortality. The risk of all-cause mortality was higher in patients with resected or resectable tumors than in those with nonresectable tumors and was higher in patients with new-onset DM than in those with longstanding DM. These observations could not be explained by confounding factors, publication bias, or undue influence by a single study.

To our knowledge, our meta-analysis is the first exclusive study of the association between DM and PC outcome, even though this topic has been investigated by many individual studies. Our results are not in accordance with a previous meta-analysis of long-term all-cause mortality in cancer patients with preexisting DM by Barone *et al.*^8^, who found that DM was associated with increased risk of mortality in all cancers (HR: 1.41; 95% CI: 1.28–1.55) but not specifically in PC (HR: 1.09; 95% CI: 0.70–1.69). The discrepancy between the two studies could be partially explained by low power (7%) of the previous study since only four studies in the PC subgroup with a population of 1,681 patients, including 477 DM patients, were analyzed. In the current review and larger-scale meta-analyses, we conducted post hoc power calculations and found that our study had 85% power in demonstrating the association between DM and cancer mortality. Furthermore, we observed that the negative effect of DM on survival occurred primarily in patients with resected or resectable pancreatic tumors (HR, 1.37; 95% CI, 1.15–1.63) but not in patients with late-stage disease (HR, 1.07; 95% CI, 0.89–1.29). These observations support the hypothesis that the previous inconsistent findings between individual studies might be partially explained by the different patient populations involved.

There were several limitations in the literature and thus in our systematic review and meta-analysis. First, studies varied in their inclusion criteria, study population, and adjustment for confounding variables, which may have led to both overestimations and underestimations of risks. Nevertheless, our sensitivity analyses, excluding studies that did not adjust for potential confounders, did not materially change the results. Residual or unknown confounding is still possible after adjusting for most relevant confounding factors. The association may not necessarily be causal as well, particularly in the observational studies^63^. Given that the start time of survival of each study differs by cancer stage (ie. tumor resection), we also performed a subgroup analysis by cancer stage (resected/resectable, unresectable and mixed stages), which confirmed the association of diabetes with poor survival in patients with resected/resectable tumor.

Second, the status of DM ascertainment varied across studies, and the duration of DM was not directly reported in some studies. Moreover, in most studies, diabetic status was based exclusively on past medical history; thus, there was a chance of misclassification, which may have led to underestimation of the number of patients with DM and of the effect of the disease.

Third, overlap in patient enrollment between some studies may have elevated their weight in the quantitative analysis. For example, the patient cohort used by Cannon *et al.*^28^ to derive and test the survival prognostic scoring model included the 209 patients from Chu *et al.*’s prior study^27^, and these patients were randomly divided into training and validation sets. In addition, two studies conducted at the same institution, i.e., Li *et al.*’s study of all-stage patients recruited between 1999 and 2008 and Sahin *et al.*’s study of patients who had undergone resection and were recruited between 1996 and 2001, may have some overlapping patients. However, we expect that the effects of this overlap on the final results of our analysis to be minimal.

The fourth limitation was that most of the articles did not report the types of anticancer and antidiabetic therapies used or their effects on outcomes. This is important because studies have shown that some therapies (e.g., surgery, adjuvant chemotherapy, and the antidiabetic drug metformin) have a more positive effect than others on cancer outcome^64^,^65^.

There are several potential explanations for the observed association between decreased survival time and DM in PC patients. First, DM may enhance tumor progression via the mechanisms of insulin resistance and inflammation, i.e., the same mechanisms that contributed to the higher risk of PC may also be responsible for the poor outcome of the disease. It has been suggested that hormonal or metabolic abnormalities, such as hyperinsulinemia or hyperglycemia, may affect tumor biology at multiple stages, including malignant transformation, growth, and metastasis^66^.

Second, PC patients with DM may be given less vigorous anticancer regimens because they generally have more contradictions to surgery, chemotherapy, and other treatments, although selection bias associated with types of surgery, chemotherapy, and radiotherapy were not seen in previous studies^27^. The metabolic abnormalities associated with DM may have an adverse effect on response to cancer treatment^67^.

Finally, the high mortality rate observed among patients with DM may partially be due to noncancerous factors, such as complications of long-term DM. However, since most PC patients die of the disease within a short period of time, it is unlikely that the complications of long-term DM would make a significant contribution to PC mortality. Only one study measured PC-specific mortality and observed a significant impact of DM^36^.

Previous studies^2^,^6^ have shown that new-onset DM, compared with longstanding DM, was associated with greater risk of PC because new-onset DM was a manifestation of subclinical PC. The main implication of our study is that DM is significantly associated with adverse outcome in PC. Subgroup analyses showed that the effect of diabetes on overall survival was associated with the cancer stages and the duration of diabetes. Our results reveal the need for further prospective studies to confirm DM as a prognostic factor and to assess the possibility of an antidiabetic regimen in the treatment of PC.

## Additional Information

**How to cite this article**: Mao, Y. *et al.* Effect of Diabetes Mellitus on Survival in Patients with Pancreatic Cancer: A Systematic Review and Meta-analysis. *Sci. Rep.* **5**, 17102; doi: 10.1038/srep17102 (2015).

## Supplementary Material

Supplementary Tables and Figures

## Figures and Tables

**Table 1:** Characteristics of 29 studies included in the meta-analysis of the effect of preexisting DM on pancreatic cancer all-cause, long-term mortality.

Study, year, country	Date of recruitment (range)	Inclusion criteria	Exclusion criteria	Patients with DM No./Total No. (%)	Age at diagnosis (y)	Male No. (%)	Follow-up time (months)	Survival (HR, 95% CI)	Adjustments
Sperti^24^, 1996, Italy	1970–1992	Pathologically confirmed; resected	NA	62/113 (54.9)	59.4 (27–81)	66 (58.4)	NA	3.02 (1.38–6.60)*	Age, stage, grade
Neoptolemos^9^, 2001, UK	1994–2000	Pathologically confirmed; resected	NA	85/541 (15.7)	60 (53–67)	324 (59.9)	10 (1–25)	1.93 (0.77–2.17) [margin positive] 1.17 (0.85–2.00) [margin negative]	None
Sperti^10^, 2003, Italy	1996–2002	Pathologically confirmed	NA	20/60 (33.3)	66.3 (48 –82)	34 (56.7)	1–35	0.63 (0.32–1.22), *P* = 0.17†	Age, sex, SUV, tumor stage, tumor grade, treatment, CA 19–9,
van de Poll–Franse^25^, 2007, The Netherlands	1995–2002	NA	NA	245/1211 (20.2)	NA	NA	NA	1.16 (1.00–1.34), *P* < 0.05	Age, sex, stage, treatment
Li^11^, 2007, USA	1999–2004	Pathologically confirmed	NA	88/378 (23.3)	NA	207 (54.8)	34 (18–90)	1.186 (0.901–1.560), *P* = 0.224	Age, sex, race, stage
Li^26^, 2009, USA	2004–2008	Pathologically confirmed	NA	221/841 (26.3)	61.7 (61.0–62.4)	496 (59.0)	22.1 (20.2–24.0)	1.29 (1.02–1.64)	Stage, resection, BMI
Chu^27^, 2010, USA	2000–2007	Pathologically confirmed; resected	Other periampullary adenocarcinomas	93/209 (44.5)‡; new-onset: 55/93 (59.1); longstanding 35/93 (37.6)	65 (37–86)	103 (49.3)*	NA	1.55 (1.02–2.35), P = 0.04; New–onset DM§ 1.75 (1.10–2.78), *P* = 0.017 Longstanding DM§ 1.30 (0.75–2.25), *P* = 0.36	Age, sex, ethnicity, BMI, Charlson, comorbidities, smoking, tumor size, node and margin, perineural and lymphovascular invasion, adjuvant therapy
McWilliams^12^, 2010, USA	2000–2009	PAC	Missing height, weight, or disease stage	472/1529 (30.9)	66.0 (58.0–74.0)||	864 (56.5)	Median 306 d	1.08 (0.95–1.22), *P* = 0.229	Age (continuous variable), sex, BMI
Olson^13^, 2010, USA	2004–2008	Over 21 years; pathologically confirmed	NA	47/475 (9.9)	63.7 ± 10.8	247 (52.0)	NA	0.97 (0.42–2.26), *P* = 0.95 [resected]; 0.78 (0.47–1.30), *P* = 0.34 [unresected]	None
Dandona^14^, 2011, USA	1995–2009	Resected	Undocumented BMI, BMI < 18.5 kg/m^2^	116/355 (32.7)	65.5 ± 10.2	192 (54.1)	32.3 (range, 0.56–51.77)	0.855 (0.650–1.124)	Age, sex, N–stage, BMI
Cannon^28^, 2011, USA	2000–2009	Resected	Margin positive, neoadjuvant chemotherapy	78/245 (31.8)¶	67.0 (58.0–74.0)	115 (46.9)	Median 4.5	1.99 (1.40–2.82), *P* < 0.001	Tumor size, node and margin
Morizane^15^, 2011, Japan	2001–2007	Pathologically confirmed; metastatic PC with gemcitabine first–line therapy	NA	171/409 (41.8)	64 (21–81)	241 (58.9)	0.4–41.3	0.997 (0.818–1.217)	None
Hartwig^29^, 2011, Germany	2001–2009	Resected	Ampullary carcinomas or carcinomas of the distal bile duct	151/1071 (14.6)	65.4 (57.9–71.1)	599 (55.9)	17 (10–31)	1.53 (1.20–1.94), *P* = 0.0005	Age, CA 19-9, TNM staging, grade, margin status
Vickers^16^, 2012, Canada	2001–2003	Pathologically confirmed; unresectable	Prior chemotherapy except for chemoradiation	175/569 (30.8)	63.9 (36.1–92.4)	298 (52.4)	Median 13.9	1.21 (0.99–1.47), *P* = 0.058	Sex, race, performance status, baseline pain intensity
Ben^30^, 2012, China	2005–2010	Histologically confirmed; resected	History of cancers, no FBG test	107/396 (27.0)#	63.1 ± 9.1	(63.5)	20 (4–62)	1.385 (1.068–1.796), *P* = 0.014	Age, sex, CA19-9, node invasion, stage, neural invasion
Inal^18^, 2012, Turkey	2005–2011	≥18 years; pathologically confirmed; locally advanced or metastatic	NA	127/406 (31.3)	63 [Gemcitabine], 57 [Gemcitabine plus Cisplatin]	273 (67.2)	NA	0.93 (0.53–1.62)	None
Sahin^31^, 2012, USA	1996–2011	Pathologically confirmed; resected	NA	144/544 (26.5)	NA	314 (57.7)	NA	1.29 (1.02–1.64), *P* = 0.036	Perineural invasion, margin status, node status, grade
Gong^43^, 2012, USA	1995–1999	PAC	NA	72/510 (14.1)	66.1 [BMI < 25 kg/m^2^],63.2 [BMI = 25–30 kg/m^2^], 62.0 [BMI ≥ 30 kg/m^2^]	278 (54.5)	10.1 y	0.85 (0.64–1.13)	Age, sex, race, education, BMI, smoking status, stage, tumor grade, tumor site, primary treatment
Barbas^21^, 2012, USA	1996–2008	Pathologically confirmed PAC; underwent pancreaticoduodenectomy	Significant missing clinicopathological data	51/203 (25.1)	<65 (47.8%); 65–74(36.4%); ≥ 75 (15.8%)	106 (52.2)	NA	1.2 (0.76–1.90), *P* = 0.42	Age, adjuvant therapy, coronary artery disease, histology, neoadjuvant therapy, resection margin, perineural Invasion, lymph node, vascular invasion
Hwang^20^, 2013, USA	2003–2010	>40 years; with a diagnostic code for PAC	Had PAC before the start of follow-up in THIN	745/3147 (23.7)**	72.3 ± 10 [T2DM], 71.2 ± 11.6 [non-T2DM]	1524 (48.4)	NA	1.02 (0.93–1.12), *P* = 0.620	Age, sex, resection, history of pancreatitis, Charlson index
Zhou^44^, 2013, China	2002–2007	Pathologically confirmed; resected; underwent pancreatectomy	NA	54/114 (47.4)	64 (31–79)	79 (69.3)	15.0 (0.2–60)	1.218 (0.765–1.941), *P* = 0.406	None
Zeiss^45^, 2013, Germany	2009–2010	>18 years; pathologically confirmed; stage III-IV; received palliative first-line gemcitabine-based chemotherapy	Adjuvant chemotherapy	16/30 (53.3)	69 (41–82)	17 (56.7)	5.8 (0.9–23.5)	1.49 (0.69–3.23), *P* = 0.31	None
Mizuno^22^, 2013, Japan	1993–2011	PAC	NA	256/540 (47.4)	67 ± 11	322 (59.6)	NA	0.91 (0.74–1.12), *P* = 0.39	Symptoms at diagnosis, PS, CA19-9, stage, treatment
Lee^23^, 2013, Korea	2007–2010	Pathologically confirmed; received an operation, chemotherapy, or chemoradiotherapy	Received only supportive care, palliative surgery; Referred from other hospitals after receiving treatment or refusing treatment	57/187 (30.5)	65 (31–86)	104 (55.6)	11.7 (2–59.5)	0.81 (0.54–1.21), *P* = 0.312	Age, sex, PS, stage, tumor site, size, CA19-9, CEA
Choi^46^, 2014, Korea	2003–2010	Pathologically confirmed PAC; gemcitabine-based palliative chemotherapy	Double primary advanced malignancies	182/345(52.8)	60.1 (20.0–84.7)	270 (63.5)	10.3 (9.5–11.1)	0.774 (0.605–0.991), *P* = 0.042	PS, disease extent, weight loss at diagnosis (BMI change ≥ 1), weight loss during chemotherapy (BMI change ≥ 1).
Toriola^47^, 2014, USA	1993–2001	Exocrine PC	Missing information on tumor stage or diabetes	62/504 (12.3)	64	273 (54.2)	NA	1.52 (1.14–2.04), *P* < 0.01 (All exocrine pancreas cancer); 1.45 (1.06–2.00), *P* = 0.02 (Excluding pancreatic cancer cases diagnosed within 3 years of enrolment); 2.31 (1.16–4.58), *P* = 0.02 (Localized); 1.17 (0.62–2.20), *P* = 0.65 (Locally advanced); 1.52 (1.04–2.24), *P* = 0.03 (Metastatic).	Age, sex, BMI, race, smoking, stage
Dong^48^, 2014, China	2009–2012	Pathologically confirmed potentially resectable PAC; consecutive patients underwent surgery	Double cancer with life-threatening phenotype; died in within 30 days after surgery	34/114 (29.8)	60 (54–67)	64 (56.1)	NA	1.820 (1.115–2.972), *P* = 0.017	Serum calcium level, histologically poorly-differentiated tumor, existence of vessel invasion
Salem^49^, 2014, USA	2010–2013	Albumin-bound paclitaxel plus gemcitabine (Gem/nab) after first-line FOLFIRINOX	NA	NA/44	55	26 (59.1)	NA	3.8 (1.0–14.3), *P* ≤ 0.05	NA
Beg,2014, USA	1995–2008	Code for PC	Missing information on DM status or follow up	1326/4728 (28.0)	67.2	4617 (97.7)	3.6 (1.3–7.4)	0.91 (0.849–0.974), *P* = 0.0065	Age, tobacco use, disease site, stage, chemotherapy, surgery

Abbreviations: CI, confidence interval; BMI, body mass index; DM, diabetes mellitus; FBG, fasting blood glucose; GFR, glomerular filtration rate; ICD-9, International Classification of Diseases, 9th revision; NA, not available; PAC, pancreatic adenocarcinoma; PC, pancreatic cancer; SUV, standardized uptake value; THIN, The Health Improvement Network.

^*^Data from Table 1 of Sperti *et al.* study.

^†^Data from Barone *et al.* study.

^‡^DM definition: For patients without documented history of DM, FBG was tested for classification.

^§^New-onset DM: disease duration preceding PDAC diagnosis date of < 24 mo; longstanding DM: disease duration preceding PDAC diagnosis date of ≥24 mo.

^||^For all patients including those missing DM status and other data.

^¶^DM definition: A past medical history of, a preoperative fasting glucose greater than 125 mg/dL, two or more outpatient random glucose levels above 199 mg/dL.

^#^DM definition: A self-reported history of DM or a fasting blood glucose level ≥125 mg/dL (6.9 mmol/L) or postprandial blood glucose level ≥11.1 mmol/L.

^**^DM definition: With a diagnostic code for T2DM.

**Table 2:** Pooled hazard ratios of all-cause mortality in pancreatic cancer patients with and without DM.

Type of estimate	Studies (estimates), No.	Total patients, No.	Patients with DM, No.	Pooled HR (95% CI)*	I^2^, %	*P*
Total	29 (33)	19818	5257	1.13 (1.04–1.22)	66.7	<0.001
Studies of full articles	28 (32)	19774	5257	1.12 (1.03–1.21)	66.4	<0.001
Adjusted for confounders						
Any confounders	23 (25)	17843	4757	1.13 (1.03–1.24)	72.5	<0.001
Age	15 (17)	14601	3667	1.08 (0.98–1.20)	66.8	<0.001
BMI	6 (8)	3948	1036	1.17 (0.98–1.39)	61.2	0.012
Stage	12 (14)	6156	1523	1.09 (0.95–1.26)	66.7	<0.001
Patient source						
Population-based	4 (6)	5372	1124	1.12 (0.95–1.32)	59.6	0.030
Clinic-based studies	25 (27)	14446	4133	1.13 (1.02–1.25)	68.6	<0.001
DM exposure type						
Primary exposure	10 (12)	11638	3110	1.21 (1.06–1.39)	78.6	<0.001
One of multiple exposures	19 (21)	8180	2147	1.07 (0.96–1.20)	54.6	0.001
Cancer Stage						
Resected or resectable	13 (14)	4473	1037	1.37 (1.15–1.63)	61.2	0.001
Unresectable	8 (9)	2214	718	1.07 (0.89–1.29)	53.6	0.028
Mixed stages	10 (10)	13131	3502	1.01 (0.93–1.10)	60.2	0.007

Abbreviations: BMI, body mass index; CI, confidence; interval; DM, diabetes mellitus; HR, hazard ratio.

^*^Estimates calculated with use of a random-effects model.

## References

[b1] Everhart J. & Wright D. Diabetes mellitus as a risk factor for pancreatic cancer. A meta-analysis. JAMA 273, 1605–9 (1995). 7745774

[b2] Huxley R., Ansary-Moghaddam A., Berrington de Gonzalez A., Barzi F. & Woodward M. Type-II diabetes and pancreatic cancer: a meta-analysis of 36 studies. Br J Cancer 92, 2076–83 (2005). 15886696 10.1038/sj.bjc.6602619 PMC2361795

[b3] Li D. & Mao Y. Diabetes as a Risk Factor of Pancreatic Cancer. Pancreapedia: Exocrine Pancreas Knowledge Base 10.3998/panc.2015.2 (2015).

[b4] Chari S. T. *et al.* Probability of pancreatic cancer following diabetes: a population-based study. Gastroenterology 129, 504–11 (2005). 16083707 10.1053/j.gastro.2005.05.007 PMC2377196

[b5] Permert J. *et al.* Is profound peripheral insulin resistance in patients with pancreatic cancer caused by a tumor-associated factor? American Journal of Surgery 165, 61–67 (1993). 8380314 10.1016/s0002-9610(05)80405-2

[b6] Gupta S. *et al.* New-onset diabetes and pancreatic cancer. Clin Gastroenterol Hepatol 4, 1366–72 quiz 1301 (2006). 16945591 10.1016/j.cgh.2006.06.024

[b7] Barone B. B. *et al.* Postoperative mortality in cancer patients with preexisting diabetes: systematic review and meta-analysis. Diabetes Care 33, 931–9 (2010). 20351229 10.2337/dc09-1721 PMC2845055

[b8] Barone B. B. *et al.* Long-term all-cause mortality in cancer patients with preexisting diabetes mellitus: a systematic review and meta-analysis. JAMA 300, 2754–64 (2008). 19088353 10.1001/jama.2008.824 PMC3093051

[b9] Neoptolemos J. P. *et al.* Influence of resection margins on survival for patients with pancreatic cancer treated by adjuvant chemoradiation and/or chemotherapy in the ESPAC-1 randomized controlled trial. Annals of Surgery 234, 758–768 (2001). 11729382 10.1097/00000658-200112000-00007 PMC1422135

[b10] Sperti C. *et al.* 18-Fluorodeoxyglucose positron emission tomography in predicting survival of patients with pancreatic carcinoma. J Gastrointest Surg 7, 953–959 (2003). 14675704 10.1016/j.gassur.2003.09.002

[b11] Li D. *et al.* Effects of base excision repair gene polymorphisms on pancreatic cancer survival. Int J Cancer 120, 1748–1754 (2007). 17230526 10.1002/ijc.22301 PMC1892183

[b12] McWilliams R. R. *et al.* Obesity adversely affects survival in pancreatic cancer patients. Cancer 116, 5054–5062 (2010). 20665496 10.1002/cncr.25465 PMC2963722

[b13] Olson S. H. *et al.* Allergies, obesity, other risk factors and survival from pancreatic cancer. Int J Cancer 127, 2412–2419 (2010). 20143395 10.1002/ijc.25240

[b14] Dandona M. *et al.* Influence of obesity and other risk factors on survival outcomes in patients undergoing pancreaticoduodenectomy for pancreatic cancer. Pancreas 40, 931–937 (2011). 21747317 10.1097/MPA.0b013e318215a9b1

[b15] Morizane C. *et al.* Construction and validation of a prognostic index for patients with metastatic pancreatic adenocarcinoma. Pancreas 40, 415–421 (2011). 21283041 10.1097/MPA.0b013e3182021376

[b16] Vickers M. M. *et al.* Comorbidity, age and overall survival in patients with advanced pancreatic cancer—Results from NCIC CTG PA.3: A phase III trial of gemcitabine plus erlotinib or placebo. European Journal of Cancer 48, 1434–1442 (2012). 22119354 10.1016/j.ejca.2011.10.035

[b17] Nakai Y. *et al.* Clinical Outcomes of Chemotherapy for Diabetic and Nondiabetic Patients With Pancreatic Cancer: Better Prognosis With Statin Use in Diabetic Patients. Pancreas 42, 202–208 (2013). 23000889 10.1097/MPA.0b013e31825de678

[b18] Inal A. *et al.* Gemcitabine alone versus combination of gemcitabine and cisplatin for the treatment of patients with locally advanced and/or metastatic pancreatic carcinoma: a retrospective analysis of multicenter study. Neoplasma 59, 297–301 (2012). 22329849 10.4149/neo_2012_038

[b19] Ganti A. K. *et al.* Predictive value of clinical features at initial presentation in pancreatic adenocarcinoma: a series of 308 cases. Med Oncol 19, 233–237 (2002). 12512917 10.1385/MO:19:4:233

[b20] Hwang A., Narayan V. & Yang Y. X. Type 2 diabetes mellitus and survival in pancreatic adenocarcinoma: A retrospective cohort study. Cancer 119, 404–410 (2013). 23292900 10.1002/cncr.27731

[b21] Barbas A. S. *et al.* Comparison of Outcomes and the Use of Multimodality Therapy in Young and Elderly People Undergoing Surgical Resection of Pancreatic Cancer. Journal of the American Geriatrics Society 60, 344–350 (2012). 22211710 10.1111/j.1532-5415.2011.03785.x

[b22] Mizuno S. *et al.* Diabetes is a useful diagnostic clue to improve the prognosis of pancreatic cancer. Pancreatology 13, 285–289 (2013). 23719602 10.1016/j.pan.2013.03.013

[b23] Lee K. J. *et al.* Serum CA 19-9 and CEA levels as a prognostic factor in pancreatic adenocarcinoma. Yonsei Medical Journal 54, 643–649 (2013). 23549809 10.3349/ymj.2013.54.3.643 PMC3635646

[b24] Sperti C., Pasquali C., Piccoli A. & Pedrazzoli S. Survival after resection for ductal adenocarcinoma of the pancreas. Br J Surg 83, 625–631 (1996). 8689203 10.1002/bjs.1800830512

[b25] Van de Poll-Franse L. V. *et al.* Less aggressive treatment and worse overall survival in cancer patients with diabetes: a large population based analysis. Int J Cancer 120, 1986–1992 (2007). 17230509 10.1002/ijc.22532

[b26] Li D., Hassan M. M. & Abbruzzese J. L. Obesity and survival among patients with pancreatic cancer: Reply. JAMA—Journal of the American Medical Association 302, 1752–1753 (2009). 10.1001/jama.2009.1510 19861663

[b27] Chu C. K. *et al.* Preoperative diabetes mellitus and long-term survival after resection of pancreatic adenocarcinoma. Ann Surg Oncol 17, 502–513 (2010). 19885697 10.1245/s10434-009-0789-6

[b28] Cannon R. M. *et al.* Multi-institutional analysis of pancreatic adenocarcinoma demonstrating the effect of diabetes status on survival after resection. Hpb 14, 228–235 (2012). 22404260 10.1111/j.1477-2574.2011.00432.x PMC3371208

[b29] Hartwig W. *et al.* Pancreatic Cancer Surgery in the New Millennium Better Prediction of Outcome. Annals of Surgery 254, 311–319 (2011). 21606835 10.1097/SLA.0b013e31821fd334

[b30] Ben Q. *et al.* Clinical profiles and long-term outcomes of patients with pancreatic ductal adenocarcinoma and diabetes mellitus. Diabetes/Metabolism Research and Reviews 28, 169–176 (2012). 22423386 10.1002/dmrr.1284

[b31] Sahin I. H. *et al.* Association of diabetes and perineural invasion in pancreatic cancer. Cancer Med 1, 357–362 (2012). 23342285 10.1002/cam4.43 PMC3544459

[b32] Coste F. *et al.* Prognostic factors in exocrine pancreas cancer. 72 patients. Revue de Medecine Interne 13, S62 (1992).

[b33] Wakasugi H., Funakoshi A. & Iguchi H. Clinical observations of pancreatic diabetes caused by pancreatic carcinoma, and survival period. Int J Clin Oncol 6, 50–54 (2001). 11706528 10.1007/pl00012080

[b34] Ragulin-Coyne E. *et al.* Pancreatic cancer following a diagnosis of new-onset diabetes: A population-based study. Hpb 13, 53–54 (2011).

[b35] Mao Y., Tao M., Jia X. & Li D. Diabetes Associated With Short Survival in Pancreatic Cancer. Journal of Clinical Oncology 33, 2120–1 (2015). 25940720 10.1200/JCO.2014.60.2896

[b36] Busaidy N. L. *et al.* Survival of resectable pancreatic cancer patients with diabetes. Journal of Clinical Oncology 24, 202s–202s (2006).

[b37] Mizuno S. *et al.* New-onset diabetes mellitus is not associated with the prognosis of pancreatic cancer. Gastroenterology 136, A390 (2009).

[b38] Yazbeck C. F. *et al.* Diabetes mellitus confers a poor prognosis on resectable pancreatic cancer patients. Diabetes 55, A207–A207 (2006).

[b39] Stroup D. F. *et al.* Meta-analysis of observational studies in epidemiology: a proposal for reporting. Meta-analysis Of Observational Studies in Epidemiology (MOOSE) group. JAMA 283, 2008–12 (2000). 10789670 10.1001/jama.283.15.2008

[b40] Von Elm E. *et al.* The Strengthening the Reporting of Observational Studies in Epidemiology (STROBE) statement: guidelines for reporting observational studies. Lancet 370, 1453–7 (2007). 18064739 10.1016/S0140-6736(07)61602-X

[b41] Higgins J. P., Thompson S. G., Deeks J. J. & Altman D. G. Measuring inconsistency in meta-analyses. BMJ 327, 557–60 (2003). 12958120 10.1136/bmj.327.7414.557 PMC192859

[b42] Duval S. & Tweedie R. Trim and fill: A simple funnel-plot-based method of testing and adjusting for publication bias in meta-analysis. Biometrics 56, 455–63 (2000). 10877304 10.1111/j.0006-341x.2000.00455.x

[b43] Gong Z. H., Holly E. A. & Bracci P. M. Obesity and survival in population-based patients with pancreatic cancer in the San Francisco Bay Area. Cancer Causes & Control 23, 1929–1937 (2012). 23015286 10.1007/s10552-012-0070-3 PMC3506392

[b44] Zhou L. *et al.* Upregulation of transgelin is an independent factor predictive of poor prognosis in patients with advanced pancreatic cancer. Cancer Sci 104, 423–430 (2013). 23331552 10.1111/cas.12107 PMC7657166

[b45] Zeiss K. *et al.* Glucose and lipid metabolism in patients with advanced pancreatic cancer receiving palliative chemotherapy. Anticancer Res 33, 287–292 (2013). 23267159

[b46] Choi Y. *et al.* The Impact of Body Mass Index Dynamics on Survival of Patients With Advanced Pancreatic Cancer Receiving Chemotherapy. Journal of Pain and Symptom Management 48, 13–25 (2014). 24321510 10.1016/j.jpainsymman.2013.08.017

[b47] Toriola A. T., Stolzenberg-Solomon R., Dalidowitz L., Linehan D. & Colditz G. Diabetes and pancreatic cancer survival: A prospective cohort-based study. British Journal of Cancer 111, 181–185 (2014). 24786605 10.1038/bjc.2014.224 PMC4090724

[b48] Dong Q. *et al.* Serum calcium level used as a prognostic predictor in patients with resectable pancreatic ductal adenocarcinoma. Clin Res Hepatol Gastroenterol 38, 639–648 (2014). 24630955 10.1016/j.clinre.2014.01.012

[b49] Salem M. E. *et al.* Albumin-bound paclitaxel plus gemcitabine after first-line FOLFIRINOX therapy in patients with pancreatic cancer. Journal of Clinical Oncology 32, e15252 (2014).

[b50] Beg M. S., Dwivedi A. K., Ahmad S. A., Ali S. & Olowokure O. Impact of diabetes mellitus on the outcome of pancreatic cancer. PLoS One 9, e98511 (2014). 24879130 10.1371/journal.pone.0098511 PMC4039482

[b51] Dehayem Y. M. *et al.* Impact of diabetes mellitus on clinical presentation and prognosis of pancreatic cancer. Ann Endocrinol (Paris) 72, 24–29 (2011). 21196000 10.1016/j.ando.2010.10.001

[b52] Partelli S. *et al.* Faecal elastase-1 is an independent predictor of survival in advanced pancreatic cancer. Digestive and Liver Disease 44, 945–951 (2012). 22749648 10.1016/j.dld.2012.05.017

[b53] Teo M. Y., McDonnell F. & McDermott R. Glycaemic status and survival in locally-advanced pancreatic adenocarcinoma. Pancreatology 12, e18 (2012).

[b54] Lee T. Y., Cheon Y. K. & Shim C. S. High hemoglobin A1C level is associated with worse survival in advanced pancreatic cancer patients with diabetes. Gastroenterology 144, S661 (2013). 10.5009/gnl.2014.8.2.205 PMC3964272 24672663

[b55] Furukawa K. *et al.* Prognostic factors of unresectable pancreatic cancer treated with nafamostat mesilate combined with gemcitabine chemotherapy. Anticancer Research 32, 5121–5126 (2012). 23155291

[b56] Esbah O. *et al.* Metformin in diabetic pancreatic cancer patients: Benefit or not-Multicenter experience. Journal of Clinical Oncology 31, e15110 (2013).

[b57] Pelucchi C. *et al.* Smoking and body mass index and survival in pancreatic cancer patients. Pancreas 43, 47–52 (2014). 24177141 10.1097/MPA.0b013e3182a7c74b

[b58] Winter J. M. *et al.* 1423 pancreaticoduodenectomies for pancreatic cancer: A single-institution experience. J Gastrointest Surg 10, 1199–1210 (2006). 17114007 10.1016/j.gassur.2006.08.018

[b59] Chu C. K. *et al.* Impact of diabetes mellitus on perioperative outcomes after resection for pancreatic adenocarcinoma. J Am Coll Surg 210, 463–73 (2010). 20347739 10.1016/j.jamcollsurg.2009.12.029

[b60] Malleo G. *et al.* Preoperative diabetes mellitus does not impact on postoperative morbidity after pancreatic resections for ductal adenocarcinoma. Pancreatology 12 (6), 589–590 (2012).

[b61] Andren-Sandberg A. & Ihse I. Factors influencing survival after total pancreatectomy in patients with pancreatic cancer. Annals of Surgery 198, 605–10 (1983). 6639161 10.1097/00000658-198311000-00008 PMC1353132

[b62] Bakkevold K. E. & Kambestad B. Morbidity and mortality after radical and palliative pancreatic cancer surgery. Risk factors influencing the short-term results. Annals of Surgery 217, 356–68 (1993). 7682052 10.1097/00000658-199304000-00007 PMC1242802

[b63] Johnson J. A., Gale E. A., Johnson J. A. & Gale E. A. M. Diabetes, insulin use, and cancer risk: are observational studies part of the solution-or part of the problem? Diabetes 59, 1129–31 (2010). 20427699 10.2337/db10-0334 PMC2857892

[b64] Bowker S. L., Yasui Y., Veugelers P. & Johnson J. A. Glucose-lowering agents and cancer mortality rates in type 2 diabetes: assessing effects of time-varying exposure. Diabetologia 53, 1631–1637 (2010). 20407744 10.1007/s00125-010-1750-8

[b65] Sadeghi N., Abbruzzese J. L., Yeung S. C. J., Hassan M. & Li D. H. Metformin Use Is Associated with Better Survival of Diabetic Patients with Pancreatic Cancer. Clinical Cancer Research 18, 2905–2912 (2012). 22465831 10.1158/1078-0432.CCR-11-2994 PMC3381457

[b66] Ding X. Z., Fehsenfeld D. M., Murphy L. O., Permert J. & Adrian T. E. Physiological concentrations of insulin augment pancreatic cancer cell proliferation and glucose utilization by activating MAP kinase, PI3 kinase and enhancing GLUT-1 expression. Pancreas 21, 310–20 (2000). 11039477 10.1097/00006676-200010000-00014

[b67] Richardson L. C. & Pollack L. A. Therapy insight: Influence of type 2 diabetes on the development, treatment and outcomes of cancer. Nat Clin Pract Oncol 2, 48–53 (2005). 16264856 10.1038/ncponc0062

