# Supplementary material for: Effect of Diabetes Mellitus on Survival in Patients with Pancreatic Cancer: A Systematic Review and Meta-analysis
Source: Sci Rep. 2015 Nov 24;5:17102. doi: 10.1038/srep17102 (PMC4656995; doi:10.1038/srep17102)
Supplement: Supplementary Tables and Figures [file srep17102-s1.doc]

**Effect of Diabetes Mellitus on Survival in Patients with Pancreatic Cancer: A Systematic Review and Meta-analysis**

**Yixiang Mao^1,2,3,4#^, Min Tao^1,4#^, Xiaoyan Jia^2#^, Hong Xu^1^, Kai Chen^1^, Hongwei Tang^2^ & Donghui Li^2^**

**Author Affiliations**: ^1^Department of Oncology, The First Affiliated Hospital to Soochow University, Suzhou, China; ^2^Department of Gastrointestinal Medical Oncology, The University of Texas MD Anderson Cancer Center, Houston, Texas, USA; ^3^Department of Medical Oncology, Fudan University Shanghai Cancer Center, Shanghai, China; ^4^Jiangsu Institute of Clinical Immunology, Suzhou, China.

**^#^These authors contributed equally to the work.**

**Correspondence authors:** Yixiang Mao, MD, PhD, Department of Oncology, The First Affiliated Hospital to Soochow University, 188 Shizi Street, Suzhou 215007, China, e-mail: [maoyix@gmail.com](mailto:maoyix@gmail.com) OR Donghui Li, PhD, Department of Gastrointestinal Medical Oncology, Unit 426, The University of Texas MD Anderson Cancer Center, 1515 Holcombe Boulevard, Houston, Texas 77030, e-mail: [dli@mdanderson.org](mailto:dli@mdanderson.org).

**Supplementary Table 1. Literatures search strategy (PubMed)**

| **Database** | **Year included** | **Search terms** |
| --- | --- | --- |
| PubMed | Inception to September 2, 2014 | pancrea* AND cancer [sb] AND (“Diabetes Mellitus”[Mesh] OR “diabetes”[All Fields] or “Insulin Resistance”[Mesh] OR “hyperglycemia”[Mesh]) AND (incidence[MeSH:noexp] OR mortality[MeSH Terms] OR follow up studies[MeSH:noexp] OR prognos*[Text Word] OR predict*[Text Word] OR course*[Text Word]) |

**Supplementary Table 2. Characteristics and main results of 12 studies not included in the meta-a**nalysis

| **Study, year, country** | **Date of recruitment (range)** | **Study source** | **Inclusion criteria** | **Patients with DM No./Total No. (%)** | **Age at diagnosis (median, y)** | **Male No. (%)** | **Survival (mo)** | **Adjustments** |
| --- | --- | --- | --- | --- | --- | --- | --- | --- |
| **Coste** [**^1^**](#_ENREF_1)**, 1992, France** | 1982-1990 | Hôpital Saint-Eloi | NA | NA/72 | 68 ± 9 | NA | adjusted Cox proportional  HR =NA (p<0.05)* | Tumor extension, FBG>6.6 mmol/L, back pain |
| **Wakasugi** [**^2^**](#_ENREF_2)**, 2001, Japan** | 1972-1998 | National Kyushu Cancer Center | Invasive ductal carcinoma | 213/401 (53.1) | NA | 254 (63.3) | Kaplan-Meier survival  log-rank test:  Non-DM Vs. New-onset DM, *P* = 0.0120*,  Non-DM Vs. Longstanding, *P* = 0.0260* | None |
| **Ganti** [**^3^**](#_ENREF_3)**, 2002, USA** | 1986-2001 | University of North Dakota–affiliated medical center in the upper midwest | Biopsy-proven | 62/308 (20.1) | Mean 70.1 (34-96) | 160 (51.9) | Kaplan-Meier survival  log-rank test: *P* = 0.7* | None |
| **Winter** [**^4^**](#_ENREF_4)**, 2006, USA** | 1970-2006 | Johns Hopkins Hospital | Underwent pancreaticoduodenectomy | 260/1175 (22.1) | 66 (32–92) | 628 (53.4) | Cox  *P* > 0.05 | Tumor size; lymph  node; resection margin;  differentiation; COPD; bile leak; adjuvant therapy |
| **Ragulin-Coyne** [**^5^**](#_ENREF_5)**, 2011, USA** | 1991-2005 | SEER-Medicare database | Code for PC | 9897/22493 (44) | NA | NA | Kaplan-Meier survival  log-rank test:  Non-DM Vs. DM, *P* = 0.0008* [non-resected pancreatic head cancers];  Non-DM Vs. DM, *P* = 0.0001* [non-resected] | None |
| **Dehayem** [**^6^**](#_ENREF_6)**, 2011, France** | 2002-2004 | The oncology unit of the University Teaching Hospital at Grenoble | Pathologically confirmed | 56/122 (45.9) | 69.1 ± 10.9 | 71 (58.2) | Death rate during follow-up:  Non-DM 44%, DM 63%, *P* = 0.047* | None |
| **Teo** [**^7^**](#_ENREF_7)**, 2012, UK** | 2005-2011 | Adelaide and Meath Hospital Incorporating National Children Hospital | Locally advanced PC | 8/21 (38.1) | 67 (42-79) | 13 (61.9) | Kaplan-Meier survival  log-rank test: *P* > 0.05* | None |
| **Furukawa** [**^8^**](#_ENREF_8)**, 2012, Japan** | 2007-2010 | The Department of Surgery, Jikei University  Hospital | Unresectable locally advanced or metastatic PC | 19/41 (46.3) | 64 (38-79) | 30 (73.2) | Kaplan-Meier survival  log-rank test: *P* = 0.9705† | None |
| **Partelli** [**^9^**](#_ENREF_9)**, 2012, Italy** | 2007-2009 | The Department of Surgery, University of Verona | Cytologically proven advanced PC | 99/194 (51.0) | 63 (IQR 55-69) | 108 (55.7) | Kaplan-Meier survival  log-rank test: *P* = 0.407* | None |
| **Claudio** [**^10^**](#_ENREF_10)**, 2013, Italy** | NA | 2 Italian case-control studies | PC | NA/648 | NA | NA | Cox  *P* > 0.05 | NA |
| **Lee** [**^11^**](#_ENREF_11)**, 2013, Korea** | 2005-2011 | Konkuk University Hospital | Patients who were first diagnosed as having advanced PC | 65/127 (51.1);  new-onset: 28/65 (43.1);  longstanding 37/65 (56.9) | NA | NA | Log-rank  DM 198 days, non-DM 263 days, *P* = 0.091 | None |
| **Esbah** [**^12^**](#_ENREF_12)**, 2013, Turkey** | 2003-2012 | Ankara Oncology Training and Research Hospital | PAC | 173/467 (37.0) | 62 (20-85) | NA | Log-rank  DM 9 months, non-DM 8 months, *P* > 0.05 | None |

**Abbreviations:** BMI, body mass index; CI, confidence interval; DM, diabetes mellitus; FBG, fasting blood glucose; NA, not available; PAC, pancreatic adenocarcinoma; SEER, the Surveillance, Epidemiology and End Results

*DM population had worse survival than did non-DM population.

†DM population had better survival than did non-DM population.

**Supplementary Table 3.** Study quality assessment

| **Reference** | **Publication type** | | **Patient source** | | **Diabetes ascertainment** | | | | **Outcome ascertainment** | | | | **Diabetes evaluated as** | | **Statistical analysis adjusted model** |
| --- | --- | --- | --- | --- | --- | --- | --- | --- | --- | --- | --- | --- | --- | --- | --- |
|  | **Journal article** | **Abstract** | **Population-based cohort** | **Clinic-based cohort** | **Medical record or medication use** | **Laboratory results** | **Questionnaire** | **Not reported** | **Registry** | **Medical record** | **Telephone/mail follow-up** | **Not reported** | **Primary exposure** | **One of multiple exposures** |  |
| ***Section A: 29 Articles Included in the meta-analysis*** | | | | | | | | | | | | | | | |
| Sperti [^13^](#_ENREF_13), 1996 | ■ |  |  | ■ | ■ |  |  |  | ■ | ■ |  |  |  | ■ | ■ |
| Neoptolemos [^14^](#_ENREF_14), 2001 | ■ |  |  | ■ | ■ |  |  |  |  | ■ |  |  |  | ■ |  |
| Sperti [^15^](#_ENREF_15), 2003 | ■ |  |  | ■ | ■ |  |  |  |  | ■ |  |  |  | ■ | ■ |
| van de Poll-Franse [^16^](#_ENREF_16), 2007 | ■ |  | ■ |  | ■ |  |  |  | ■ | ■ |  |  | ■ |  | ■ |
| Li [^17^](#_ENREF_17), 2007 | ■ |  |  | ■ | ■ |  | ■ |  | ■ | ■ |  |  |  | ■ | ■ |
| Li [^18^](#_ENREF_18), 2009 | ■ |  |  | ■ | ■ |  |  |  | ■ | ■ |  |  |  | ■ | ■ |
| Chu [^19^](#_ENREF_19), 2010 | ■ |  |  | ■ | ■ | ■ |  |  | ■ | ■ | ■ |  | ■ |  | ■ |
| McWilliams [^20^](#_ENREF_20), 2010 | ■ |  |  | ■ | ■ |  | ■ |  | ■ | ■ | ■ |  |  | ■ | ■ |
| Olson [^21^](#_ENREF_21), 2010 | ■ |  |  | ■ | ■ |  |  |  |  | ■ |  |  |  | ■ |  |
| Dandona [^22^](#_ENREF_22), 2011 | ■ |  |  | ■ | ■ |  |  |  | ■ | ■ |  |  |  | ■ | ■ |
| Cannon [^23^](#_ENREF_23), 2011 | ■ |  |  | ■ | ■ | ■ |  |  |  | ■ |  |  | ■ |  | ■ |
| Morizane [^24^](#_ENREF_24), 2011 | ■ |  |  | ■ | ■ |  |  |  |  |  |  | ■ |  | ■ |  |
| Hartwig [^25^](#_ENREF_25), 2011 | ■ |  |  | ■ | ■ |  |  |  |  | ■ | ■ |  |  | ■ | ■ |
| Vickers [^26^](#_ENREF_26), 2012 | ■ |  |  | ■ | ■ |  |  |  |  | ■ |  |  |  | ■ | ■ |
| Ben [^27^](#_ENREF_27), 2012 | ■ |  |  | ■ | ■ |  | ■ |  | ■ | ■ | ■ |  | ■ |  | ■ |
| Inal [^28^](#_ENREF_28), 2012 | ■ |  |  | ■ |  |  |  | ■ |  |  |  | ■ |  | ■ |  |
| Sahin [^29^](#_ENREF_29), 2012 | ■ |  |  | ■ | ■ | ■ | ■ |  | ■ | ■ |  |  | ■ |  | ■ |
| Gong [^30^](#_ENREF_30), 2012 | ■ |  | ■ |  | ■ |  | ■ |  | ■ |  |  |  |  | ■ | ■ |
| Barbas [^31^](#_ENREF_31), 2012 | ■ |  |  | ■ | ■ |  |  |  |  |  |  | ■ |  | ■ | ■ |
| Hwang [^32^](#_ENREF_32), 2013 | ■ |  | ■ |  | ■ |  |  |  |  | ■ |  |  | ■ |  | ■ |
| Zhou [^33^](#_ENREF_33), 2013 | ■ |  |  | ■ | ■ |  |  |  |  |  |  | ■ | ■ |  |  |
| Zeiss [^34^](#_ENREF_34), 2013 | ■ |  |  | ■ |  |  |  | ■ |  |  |  | ■ |  | ■ |  |
| Mizuno [^35^](#_ENREF_35), 2013 | ■ |  |  | ■ | ■ |  |  |  |  |  |  | ■ | ■ |  | ■ |
| Lee [^36^](#_ENREF_36), 2013 | ■ |  |  | ■ | ■ |  |  |  |  |  |  | ■ |  | ■ | ■ |
| Choi [^37^](#_ENREF_37), 2014 | ■ |  |  | ■ | ■ | ■ |  |  |  |  |  | ■ |  | ■ | ■ |
| Toriola [^38^](#_ENREF_38), 2014 | ■ |  | ■ |  |  |  | ■ |  | ■ |  |  |  | ■ |  | ■ |
| Dong [^39^](#_ENREF_39), 2014 | ■ |  |  | ■ |  |  |  | ■ | ■ | ■ | ■ |  |  | ■ | ■ |
| Salem [^40^](#_ENREF_40), 2014 |  | ■ |  | ■ |  |  |  | ■ |  |  |  | ■ |  | ■ | ■ |
| Beg [^41^](#_ENREF_41),2014 | ■ |  |  | ■ |  |  |  | ■ |  |  |  | ■ | ■ |  | ■ |
| ***Section B: 12 Articles Included in the qualitative review but not in the meta-analysis*** | | | | | | | | | | | | | | | |
| Coste [^1^](#_ENREF_1), 1992 |  | ■ |  | ■ |  |  |  | ■ |  |  |  | ■ |  | ■ | ■ |
| Wakasugi[^2^](#_ENREF_2), 2001 | ■ |  |  | ■ | ■ | ■ |  |  |  |  |  | ■ | ■ |  |  |
| Ganti [^3^](#_ENREF_3), 2002 | ■ |  |  | ■ | ■ |  |  |  |  |  |  | ■ |  | ■ |  |
| Winter [^4^](#_ENREF_4), 2006 | ■ |  |  | ■ | ■ |  |  |  |  |  |  | ■ |  | ■ | ■ |
| Ragulin-Coyne [^5^](#_ENREF_5), 2011 |  | ■ | ■ |  | ■ |  |  |  | ■ |  |  |  | ■ |  |  |
| Dehayem [^6^](#_ENREF_6), 2011 | ■ |  |  | ■ | ■ | ■ |  |  |  | ■ | ■ |  | ■ |  |  |
| Teo [^7^](#_ENREF_7), 2012 |  | ■ |  | ■ | ■ | ■ |  |  |  |  |  | ■ | ■ |  |  |
| Furukawa [^8^](#_ENREF_8), 2012 | ■ |  |  | ■ |  |  |  | ■ |  |  |  | ■ |  | ■ |  |
| Partelli [^9^](#_ENREF_9), 2012 | ■ |  |  | ■ |  |  |  | ■ |  |  |  | ■ |  | ■ |  |
| Claudio [^10^](#_ENREF_10), 2013 |  | ■ |  | ■ | ■ |  | ■ |  |  |  |  | ■ |  | ■ | ■ |
| Esbah [^12^](#_ENREF_12), 2013 |  | ■ |  | ■ | ■ |  |  |  |  |  |  | ■ | ■ |  |  |
| Lee [^11^](#_ENREF_11), 2013 |  | ■ |  | ■ | ■ |  |  |  |  |  |  | ■ | ■ |  |  |

■ Present in study.

**Supplementary Figure 1. Begg’s Plot of 33 estimates Included in the meta-analysis of the effect of preexisting DM on long-term, all-cause mortality (*P* = 0.14)**


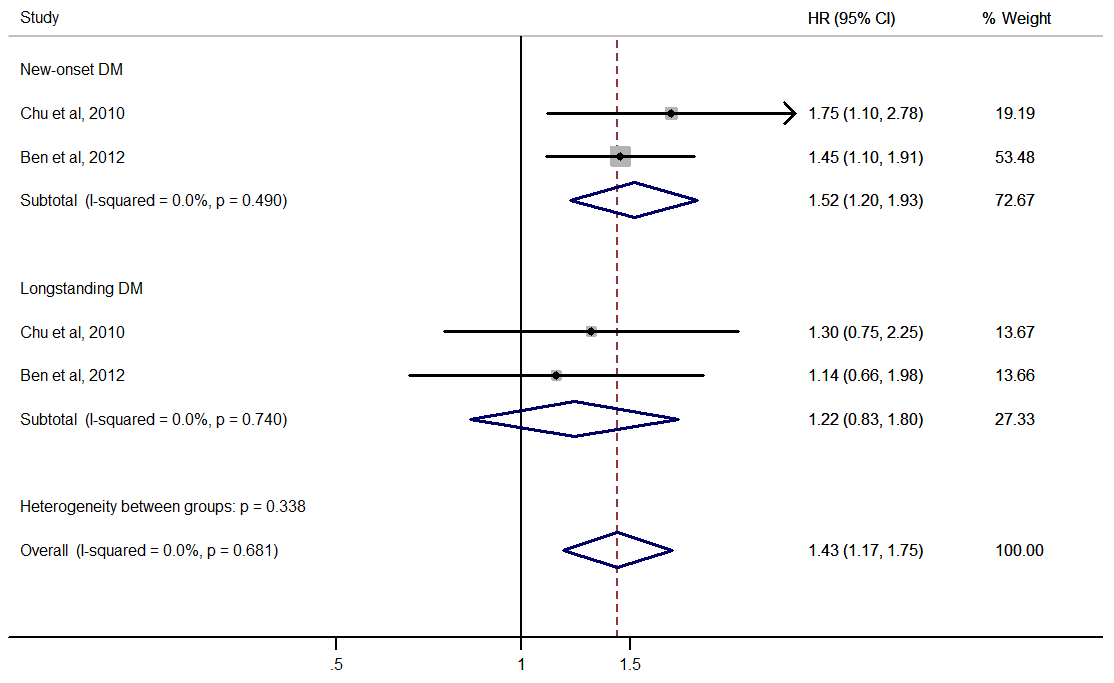


**Supplementary Figure 2. Meta-analysis of the duration of DM on all-cause mortality in patients with PC**

Abbreviations: CI, confidence interval; HR, hazard ratio.

New-onset DM: disease duration preceding PC diagnosis date of <24 mo; longstanding DM: disease duration preceding PC diagnosis date of ≥24 mo.

Squares indicate relative risk in each study. The square size is proportional to the weight of the corresponding study in the meta-analysis; the length of the horizontal lines represents the 95% CI. The unshaded diamond indicates the pooled relative risk and 95% CI.


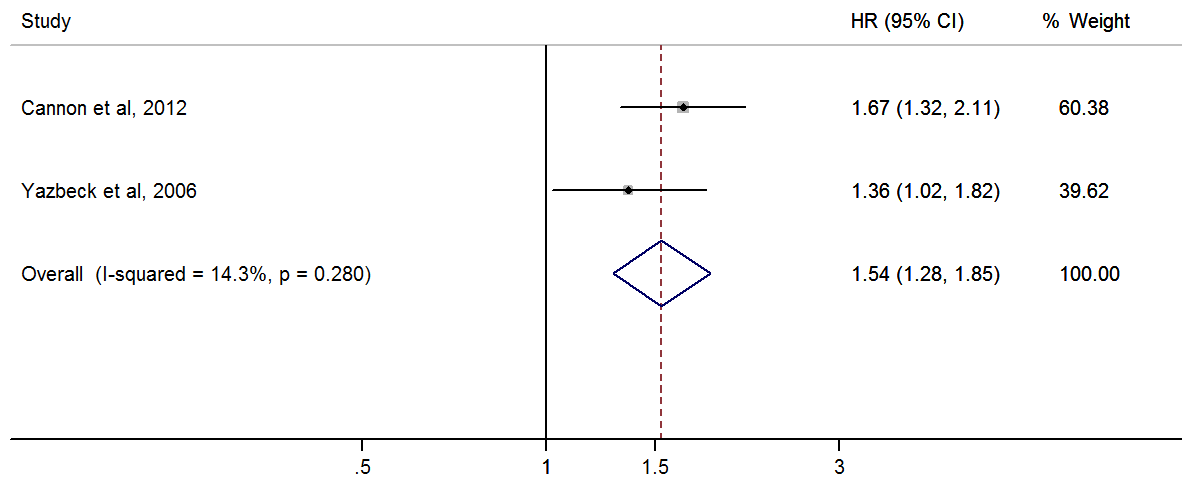


**Supplementary Figure 3. Meta-analysis of the effect of DM on DFS in patients with PC**

Abbreviations: CI, confidence interval; HR, hazard ratio.

Squares indicate relative risk in each study. The square size is proportional to the weight of the corresponding study in the meta-analysis; the length of the horizontal lines represents the 95% CI. The unshaded diamond indicates the pooled relative risk and 95% CI.

**References**

1. Coste, F. *et al.* Prognostic factors in exocrine pancreas cancer. 72 patients. *Revue de Medecine Interne* **13**, S62 (1992).

2. Wakasugi, H., Funakoshi, A. & Iguchi, H. Clinical observations of pancreatic diabetes caused by pancreatic carcinoma, and survival period. *Int J Clin Oncol* **6**, 50-54 (2001).

3. Ganti, A.K. *et al.* Predictive value of clinical features at initial presentation in pancreatic adenocarcinoma: a series of 308 cases. *Med Oncol* **19**, 233-237 (2002).

4. Winter, J.M. *et al.* 1423 pancreaticoduodenectomies for pancreatic cancer: A single-institution experience. *J Gastrointest Surg* **10**, 1199-1210 (2006).

5. Ragulin-Coyne, E. *et al.* Pancreatic cancer following a diagnosis of new-onset diabetes: A population-based study. *Hpb* **13**, 53-54 (2011).

6. Dehayem, Y.M. *et al.* Impact of diabetes mellitus on clinical presentation and prognosis of pancreatic cancer. *Ann Endocrinol (Paris)* **72**, 24-29 (2011).

7. Teo, M.Y., McDonnell, F. & McDermott, R. Glycaemic status and survival in locally-advanced pancreatic adenocarcinoma. *Pancreatology* **12**, e18 (2012).

8. Furukawa, K. *et al.* Prognostic factors of unresectable pancreatic cancer treated with nafamostat mesilate combined with gemcitabine chemotherapy. *Anticancer Research* **32**, 5121-5126 (2012).

9. Partelli, S. *et al.* Faecal elastase-1 is an independent predictor of survival in advanced pancreatic cancer. *Digestive and Liver Disease* **44**, 945-951 (2012).

10. Pelucchi, C. *et al.* Smoking and body mass index and survival in pancreatic cancer patients. *Pancreas* **43**, 47-52 (2014).

11. Lee, T.Y., Cheon, Y.K. & Shim, C.S. High hemoglobin A1C level is associated with worse survival in advanced pancreatic cancer patients with diabetes. *Gastroenterology* **144**, S661 (2013).

12. Esbah, O. *et al.* Metformin in diabetic pancreatic cancer patients: Benefit or not-Multicenter experience. *Journal of Clinical Oncology* **31**, e15110 (2013).

13. Sperti, C., Pasquali, C., Piccoli, A. & Pedrazzoli, S. Survival after resection for ductal adenocarcinoma of the pancreas. *Br J Surg* **83**, 625-631 (1996).

14. Neoptolemos, J.P. *et al.* Influence of resection margins on survival for patients with pancreatic cancer treated by adjuvant chemoradiation and/or chemotherapy in the ESPAC-1 randomized controlled trial. *Annals of Surgery* **234**, 758-768 (2001).

15. Sperti, C. *et al.* 18-Fluorodeoxyglucose positron emission tomography in predicting survival of patients with pancreatic carcinoma. *J Gastrointest Surg* **7**, 953-959 (2003).

16. van de Poll-Franse, L.V. *et al.* Less aggressive treatment and worse overall survival in cancer patients with diabetes: a large population based analysis. *Int J Cancer* **120**, 1986-1992 (2007).

17. Li, D. *et al.* Effects of base excision repair gene polymorphisms on pancreatic cancer survival. *Int J Cancer* **120**, 1748-1754 (2007).

18. Li, D., Hassan, M.M. & Abbruzzese, J.L. Obesity and survival among patients with pancreatic cancer: Reply. *JAMA - Journal of the American Medical Association* **302**, 1752-1753 (2009).

19. Chu, C.K. *et al.* Preoperative diabetes mellitus and long-term survival after resection of pancreatic adenocarcinoma. *Ann Surg Oncol* **17**, 502-513 (2010).

20. McWilliams, R.R. *et al.* Obesity adversely affects survival in pancreatic cancer patients. *Cancer* **116**, 5054-5062 (2010).

21. Olson, S.H. *et al.* Allergies, obesity, other risk factors and survival from pancreatic cancer. *Int J Cancer* **127**, 2412-2419 (2010).

22. Dandona, M. *et al.* Influence of obesity and other risk factors on survival outcomes in patients undergoing pancreaticoduodenectomy for pancreatic cancer. *Pancreas* **40**, 931-937 (2011).

23. Cannon, R.M. *et al.* Multi-institutional analysis of pancreatic adenocarcinoma demonstrating the effect of diabetes status on survival after resection. *Hpb* **14**, 228-235 (2012).

24. Morizane, C. *et al.* Construction and validation of a prognostic index for patients with metastatic pancreatic adenocarcinoma. *Pancreas* **40**, 415-421 (2011).

25. Hartwig, W. *et al.* Pancreatic Cancer Surgery in the New Millennium Better Prediction of Outcome. *Annals of Surgery* **254**, 311-319 (2011).

26. Vickers, M.M. *et al.* Comorbidity, age and overall survival in patients with advanced pancreatic cancer - Results from NCIC CTG PA.3: A phase III trial of gemcitabine plus erlotinib or placebo. *European Journal of Cancer* **48**, 1434-1442 (2012).

27. Ben, Q. *et al.* Clinical profiles and long-term outcomes of patients with pancreatic ductal adenocarcinoma and diabetes mellitus. *Diabetes/Metabolism Research and Reviews* **28**, 169-176 (2012).

28. Inal, A. *et al.* Gemcitabine alone versus combination of gemcitabine and cisplatin for the treatment of patients with locally advanced and/or metastatic pancreatic carcinoma: a retrospective analysis of multicenter study. *Neoplasma* **59**, 297-301 (2012).

29. Sahin, I.H. *et al.* Association of diabetes and perineural invasion in pancreatic cancer. *Cancer Med* **1**, 357-362 (2012).

30. Gong, Z.H., Holly, E.A. & Bracci, P.M. Obesity and survival in population-based patients with pancreatic cancer in the San Francisco Bay Area. *Cancer Causes & Control* **23**, 1929-1937 (2012).

31. Barbas, A.S. *et al.* Comparison of Outcomes and the Use of Multimodality Therapy in Young and Elderly People Undergoing Surgical Resection of Pancreatic Cancer. *Journal of the American Geriatrics Society* **60**, 344-350 (2012).

32. Hwang, A., Narayan, V. & Yang, Y.X. Type 2 diabetes mellitus and survival in pancreatic adenocarcinoma: A retrospective cohort study. *Cancer* **119**, 404-410 (2013).

33. Zhou, L. *et al.* Upregulation of transgelin is an independent factor predictive of poor prognosis in patients with advanced pancreatic cancer. *Cancer Sci* **104**, 423-430 (2013).

34. Zeiss, K. *et al.* Glucose and lipid metabolism in patients with advanced pancreatic cancer receiving palliative chemotherapy. *Anticancer Res* **33**, 287-292 (2013).

35. Mizuno, S. *et al.* Diabetes is a useful diagnostic clue to improve the prognosis of pancreatic cancer. *Pancreatology* **13**, 285-289 (2013).

36. Lee, K.J. *et al.* Serum CA 19-9 and CEA levels as a prognostic factor in pancreatic adenocarcinoma. *Yonsei Medical Journal* **54**, 643-649 (2013).

37. Choi, Y. *et al.* The Impact of Body Mass Index Dynamics on Survival of Patients With Advanced Pancreatic Cancer Receiving Chemotherapy. *Journal of Pain and Symptom Management* **48**, 13-25 (2014).

38. Toriola, A.T., Stolzenberg-Solomon, R., Dalidowitz, L., Linehan, D. & Colditz, G. Diabetes and pancreatic cancer survival: A prospective cohort-based study. *British Journal of Cancer* **111**, 181-185 (2014).

39. Dong, Q. *et al.* Serum calcium level used as a prognostic predictor in patients with resectable pancreatic ductal adenocarcinoma. *Clin Res Hepatol Gastroenterol* **38**, 639-648 (2014).

40. Salem, M.E. *et al.* Albumin-bound paclitaxel plus gemcitabine after first-line FOLFIRINOX therapy in patients with pancreatic cancer. *Journal of Clinical Oncology* **32**, e15252 (2014).

41. Beg, M.S., Dwivedi, A.K., Ahmad, S.A., Ali, S. & Olowokure, O. Impact of diabetes mellitus on the outcome of pancreatic cancer. *PLoS One* **9**, e98511 (2014).
